# Climate change and sugarcane expansion increase Hantavirus infection risk

**DOI:** 10.1371/journal.pntd.0005705

**Published:** 2017-07-20

**Authors:** Paula Ribeiro Prist, María Uriarte, Katia Fernandes, Jean Paul Metzger

**Affiliations:** 1 Department of Ecology, Bioscience Institute, University of São Paulo, São Paulo, São Paulo, Brazil; 2 Department of Ecology, Evolution & Environmental Biology, Columbia University, New York, New York, United States of America; 3 International Research Institute for Climate and Society; Earth Institute; Columbia University, Palisades, New York, United States of America; University of Minnesota, UNITED STATES

## Abstract

Hantavirus Cardiopulmonary Syndrome (HCPS) is a disease caused by Hantavirus, which is highly virulent for humans. High temperatures and conversion of native vegetation to agriculture, particularly sugarcane cultivation can alter abundance of rodent generalist species that serve as the principal reservoir host for HCPS, but our understanding of the compound effects of land use and climate on HCPS incidence remains limited, particularly in tropical regions. Here we rely on a Bayesian model to fill this research gap and to predict the effects of sugarcane expansion and expected changes in temperature on Hantavirus infection risk in the state of São Paulo, Brazil. The sugarcane expansion scenario was based on historical data between 2000 and 2010 combined with an agro-environment zoning guideline for the sugar and ethanol industry. Future evolution of temperature anomalies was derived using 32 general circulation models from scenarios RCP4.5 and RCP8.5 (Representative greenhouse gases Concentration Pathways adopted by IPCC). Currently, the state of São Paulo has an average Hantavirus risk of 1.3%, with 6% of the 645 municipalities of the state being classified as high risk (HCPS risk ≥ 5%). Our results indicate that sugarcane expansion alone will increase average HCPS risk to 1.5%, placing 20% more people at HCPS risk. Temperature anomalies alone increase HCPS risk even more (1.6% for RCP4.5 and 1.7%, for RCP8.5), and place 31% and 34% more people at risk. Combined sugarcane and temperature increases led to the same predictions as scenarios that only included temperature. Our results demonstrate that climate change effects are likely to be more severe than those from sugarcane expansion. Forecasting disease is critical for the timely and efficient planning of operational control programs that can address the expected effects of sugarcane expansion and climate change on HCPS infection risk. The predicted spatial location of HCPS infection risks obtained here can be used to prioritize management actions and develop educational campaigns.

## Author summary

Hantavirus, hosted by rodent species, causes HCPS, a disease with a 50% mortality rate in humans. The conversion of native vegetation to sugarcane increases the abundance of hantavirus reservoir rodent species, augmenting disease risk. Additionally, temperature also has positive effects on disease risk because it affects rodent population and the time the virus remains infectious in the environment. Here we evaluate the impacts of climate change and sugarcane expansion on HCPS risk. Expansion of sugarcane increases average HCPS risk placing 20% more people at risk for acquiring HCPS than under current conditions. Temperature anomalies increase HCPS risk even more and place 31% and 34% more people at risk (RCP4.5 and RCP8.5, respectively). Our results confirm the impacts of climate change and agriculture expansion on disease risk and highlight the need for timely and efficient planning of operational control programs in order to avoid disease propagation in the future.

## Introduction

Global average temperatures are projected to increase between 1.7 and 4.8°C by the end of this century [1,2], with potential effects on human health, including mortality from extreme heat and cold, and changes in the ecology of infectious diseases [3–5]. Climatic variability and extreme weather events have profound impacts on infectious diseases since fluctuations in temperature and precipitation influence both infectious agents (such as protozoa, bacteria, and viruses) and population dynamics of their vectors (such as mosquitoes, ticks, and rodents) [3, 6–8]. Outbreaks of some diseases such as Ross River virus disease [9], malaria [10], meningitis [11] and Hantavirus Cardiopulmonary Syndrome (HCPS) [12] have been associated with climate anomalies.

At the same time, increasing evidence suggests that land cover and land use change affect disease incidence by altering the interactions, abundance, and movement patterns of hosts, vectors, and people [13,14]. For instance, outbreaks of Hantavirus, Lyme disease and tick-borne encephalitis have been associated not only with climate-related changes in the density of host rodent and tick populations [15–17], but also with shifts in the extent and type of land use [17–23].

Hantavirus (*Bunyaviridae*) is a virus transmitted by small mammals [17] which causes two syndromes in humans: Hantavirus Cardiopulmonary Syndrome (HCPS), restricted to the Americas, and hemorrhagic fever with renal syndrome (HFRS) present in Eurasia and Africa [24, 25]. HCPS was first identified in 1993 in both the United States and Brazil [26, 27] and exhibits lethality rates as high as 50% [26, 28]. Unlike HFRS, a vaccine is not available for HCPS. Transmission to humans occurs through inhalation of the aerosolized form present in the urine, saliva and feces of infected rodents [29–31].

Climate conditions can influence Hantavirus host population abundance and disease transmission dynamics [32]. A number of studies in arid and semi-arid region of the U.S. have uncovered a positive association between precipitation, population size of rodent hosts and prevalence of Hantavirus [33–36]. Anomalously high precipitation increases vegetation growth, boosting rodent densities and enhancing the probability of encounters between humans and infected rodents and consequently Hantavirus transmission [12, 37]. Temperature can influence rodent abundance and disease risk by altering vegetation growth [38], reproduction and survival rates of small rodents [38–40], as well as the time the virus remains infectious in the environment [41]. The capacity of Hantavirus to survive outside its host plays a critical role in transmission dynamics [41]. High temperatures have been associated with more frequent Hantavirus outbreaks [16, 37, 42, 43], most likely because high temperature leads to greater aerosolization of the virus and higher rates of inhalation by both humans and rodents [29, 31]. There is evidence that variation in temperature, but not precipitation, affect HCPS risk in Brazil [32].

Sugarcane plantations may be also associated with increases in Hantavirus infection risk [19, 32, 44]. Experimental studies have shown that small mammal populations are frequently food-limited [45]; thus the presence of an abundant, highly nutritious food resource, such as sugarcane, with yields as high as 120 tons·year·ha−1 [46], might allow the increase and maintenance of large populations of these species, relative to other land uses, either natural or agricultural [19, 47, 48]. Furthermore, sugarcane offers protective cover for feeding, burrowing and breeding activities, throughout the year [49].

Many developing countries are expanding sugarcane plantation areas to produce biofuel, as a strategy to reduce their dependence on petroleum, to increase opportunities for the agricultural sector, and to mitigate global warming [50]. In Brazil, the creation of the pro-alcohol program, developed to replace a significant percentage of fossil-fuel consumption with ethanol produced from sugarcane [51], was triggered by an increase of 428% in oil prices in 1973 [52]. This program and the recent interest in alternative energy sources have fostered an expansion in the extent of sugarcane cultivation, making the country the world’s leader in ethanol production [53] and sugarcane (~ 490 million tons per year) exports [54]. The majority of this production (~74%) comes from the southeastern region, with the state of São Paulo producing 60% of the total yield [55, 56].

The combined consequences of bio-energy expansion and climate variability and change on Hantavirus infection risk remain unexamined. Understanding how these factors impact infectious disease risk is essential to fully evaluate the actual costs of the biofuel programs and is critical for timely and efficient planning of operational control programs. In this paper we analyze how sugarcane expansion and temperature changes under two climate scenarios can potentially influence HCPS risk in the state of São Paulo by 2050. To do so, we relied on a baseline model we previously developed [32], which evaluated the effects of landscape, climate and social predictors, including historical climate and sugarcane as predictor variables, on Hantavirus risk between 1993 and 2012. In this paper, we used climate and sugarcane data derived from various scenarios (see methods section) to test the independent and combined effect of these two factors on HCPS risk. We hypothesize that HCPS incidence will show an increase under all scenarios because both changes in climate and sugarcane expansion are expected to increase HCPS risk through their positive effects on rodent abundance and virus survival and aerosolization, and that their combined effect can exacerbate their individual impacts.

## Methods

### Study area

**Fig 1:**
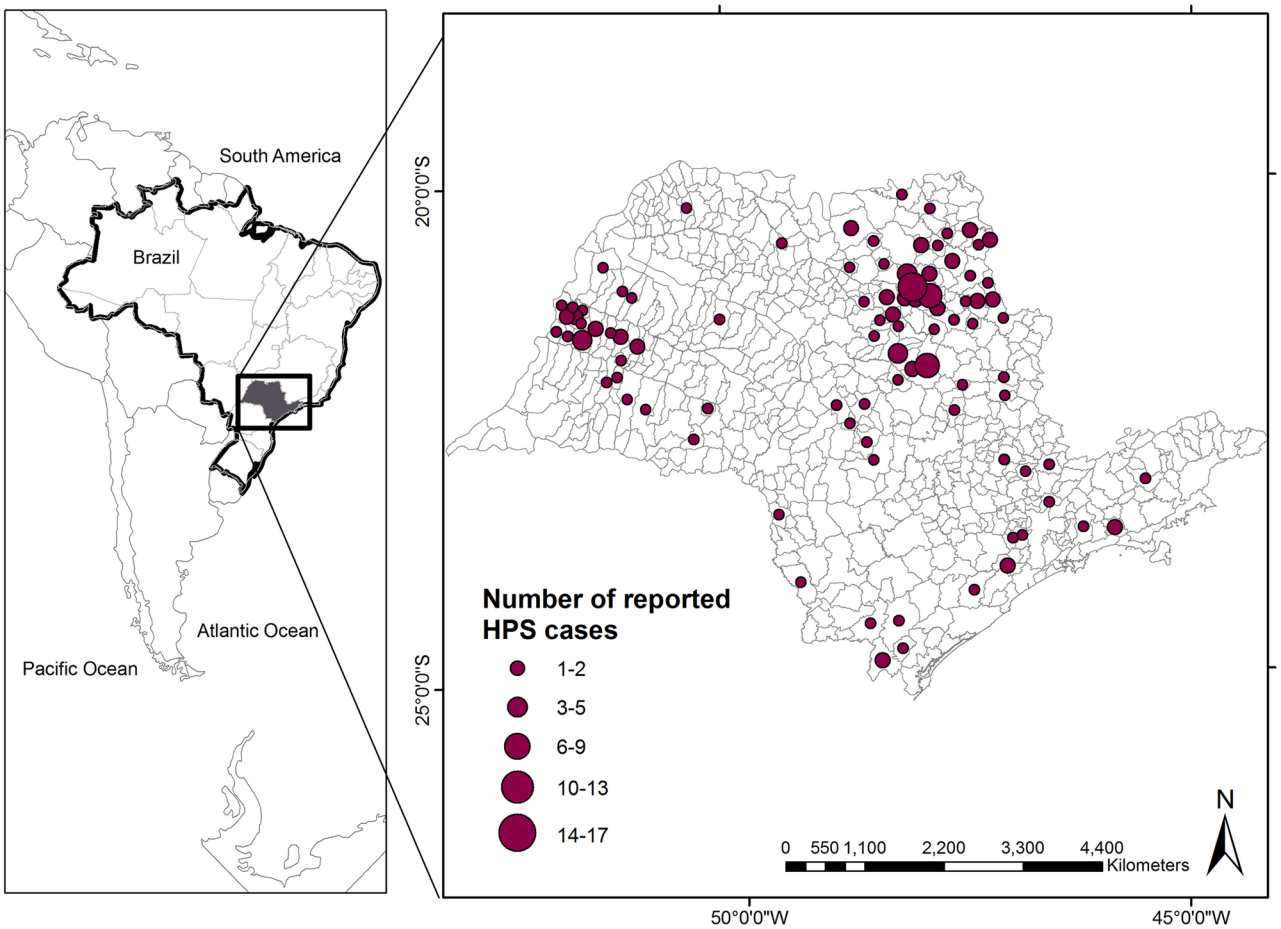
State of São Paulo. Number of HCPS cases reported between 1993 and 2012 in the state of São Paulo (Brazil).

We focused our analyses on the state of São Paulo, the wealthiest Brazilian state, where HCPS was first identified in 1993 and where the risk of disease increase is particularly high, due to both sugarcane expansion and climate change. São Paulo state is located in southeastern Brazil, in an area of approximately 248,210 km^2^ (Fig 1), and has a population of about 42 million, representing 21% of the Brazilian population [57].

### Baseline model

#### Disease and social data

We collected HCPS incidence data (number of HCPS cases per year) at the municipality level and therefore considered the 645 municipalities that compose the state as our sampling units (Fig 1). The number of reported annual HCPS cases in each municipality for the period between 1993 and 2012 is available from the Center for Epidemiological Surveillance of the State of São Paulo—CVE-SP (1993–2012), and the Health Portal SUS: http://www.saude.sp.gov.br/cve-centro-de-vigilancia-epidemiologica-prof.-alexandre-vranjac/areas-de-vigilancia/doencas-de-transmissao-por-vetores-e-zoonoses/agravos/hantavirose/dados-estatisticos. The CVE-SP compiles this information because Hantavirus infection requires mandatory notification to the local health authorities in Brazil. The data is provided by every hospital in the state. CVE-SP data include only cases recorded with clinical symptoms that have been confirmed by laboratory analysis (antibody positive). Since the majority of cases were zeroes (98.71%) or ones (1.06%), we transformed the data into a binary variable, presence/absence of HCPS per year and per municipality.

Epidemiologic data indicate that more than 70% of infected people work or live in agricultural areas, and around 93% are men over the age of twenty [58, 59]. Because the available data is relatively coarse with respect to age categories, we used the number of rural men older than 14 years in each municipality (the closest to the 20 years indicated by epidemiological data) as the population at risk for HCPS. This information was used as a predictor variable and was extracted from the Brazilian National Institute of Geography and Statistics—IBGE website (www.ibge.gov.br) and is available for 1996 and 2006. For the analyses presented here, we used population data from 1996 to predict disease incidence from 1993 to 2001, and data from 2006 to predict disease incidence from 2002 to 2012 (S1 Table).

A number of socio-economic variables can contribute to a better understanding of HCPS transmission. The human development index (HDI) is a summary measure of key dimensions of human development and can be a suitable metric of the socio-economic factors that influence HCPS risk [32]. Therefore, HDI was also included as a covariate in the model, and was extracted at municipality level from IBGE (accessible at www.ibge.gov.br), being available for 1991, 2000 and 2010. We used HDI data from 1991 to predict disease incidence from 1993 to 1998, data from 2000 to predict disease incidence from 1999 to 2005 and from 2010 to predict disease incidence from 2006 to 2012.

#### Landscape composition and configuration metrics

We used the São Paulo state forest inventory maps (i.e. habitat cover classification map—http://iflorestal.sp.gov.br/) for 2000 and 2010 to calculate two landscape metrics (percentage of cover and density of fragments) for the native vegetation cover of each municipality. Native vegetation cover data includes the two biomes of the state, the Atlantic forest [60] and the Cerrado [61]. We performed the landscape analyses in ArcGis 10.0 and Fragstats 4.2. To match this information with available disease data, we used metrics extracted from the 2000 native vegetation cover map as covariates for the incidence model for 1993–2001, and metrics extracted from the 2010 map as covariates for the 2002–2012 period.

Since the1990s, rates of native vegetation loss have been dropping dramatically, and an increase in the rates of natural regeneration has resulted in a small increase in native vegetation cover in the state of São Paulo [62–64]. Since native habitat loss appears to have leveled off, we used the 2010 landscape metrics in evaluating future HCPS risk scenarios.

The proportion of sugarcane cultivated in each municipality was obtained from the agricultural census of the Institute of Agricultural Economics (www.iea.sp.gov.br) for the years 1993 to 2012. For the analyses presented here, we used annual agricultural data (1993 to 2012) to predict annual disease incidence from 1993 to 2012.

#### Current climatic variables

Current temperature and precipitation data, used in the baseline model, were obtained from the International Research Institute for Climate and Society (IRI) Data Library system (http://iridl.ldeo.columbia.edu/index.html). For more details, see [32].

### Future scenarios

#### Sugarcane expansion

Our sugarcane expansion scenario combined data from past expansion of sugarcane with an agro-environment zoning developed for the sugar and ethanol industry (AZA, http://www.ambiente.sp.gov.br). This zoning was created to minimize social and environment impacts of sugarcane [65] and to regulate the expansion of this crop within the state [66]. We considered that sugarcane expansion in each municipality will (i) occur in the same proportion to what occurred in the last ten years (2000–2010; i.e. we are considering a *business as usual scenario*), (ii) that this expansion will occur only in pasturelands, since this is the most common transition [54, 56, 67, 68], and (iii) the expansion will occur only in three zones considered as suitable for expansion according to the AZA (S1 Fig).

Percentages of suitable and unsuitable areas for sugarcane in 2012 (the last year of our baseline model) for each municipality were extracted from the AZA map using ArcGis 10.0 and Fragstats 4.2. Percentages of pasture and sugarcane, for each municipality, were extracted from the agricultural census of the Institute of Agricultural Economics. To calculate the proportion of sugarcane expansion of the last ten years, we extracted the percentage of sugarcane for each municipality from the agricultural census of the Institute of Agricultural Economics, for the years 2000 and 2010. The difference between these two percentages was considered as the 10-year sugarcane expansion, and used as the maximum value (in percentage) of expansion for each municipality. This expansion was projected respecting the amount of suitable areas according the AZA determination and the amount of pasture, and therefore, it did not always reach the maximum value.

#### Climate change scenarios

The United Nations Intergovernmental Panel on Climate Change [69] defines future climate change scenarios in terms of representative concentration pathways (RCPs). The RCPs consider mitigation scenarios that assume policy actions will be taken to achieve certain greenhouse gases (GHG) emission targets [69] consistent with a wide range of possible changes in future anthropogenic GHG emissions [1]https://en.wikipedia.org/wiki/Representative_Concentration_Pathways#cite_note-collins_rcp_ghg_emissions-3. Radiative forcing and mitigation policies are different for each RCP scenario. In the highest GHG emission scenario, RCP8.5, radiative forcing increases throughout the 21^st^ century before reaching a level of about 8.5 W m−2 at the end of the century. In the two intermediate scenarios, RCP4.5 and RCP6.0, radiative forcing stabilizes without overshoot at a level of 4.5 Wm-2 after 2100, while in the peak-and-decay scenario, RCP2.6, radiative forcing reaches a maximum near the middle of the 21^st^ century before decreasing to an eventual nominal level of 2.6 W m−2 [69, 70]. Global average temperatures show a similar increase of approximately 0.3–0.7°C for the period of 2016–2035 in all four scenarios. After this period, the magnitude of the projected climate change is substantially affected by the choice of the scenario, with the highest global temperature increase estimated under RCP8.5 [71].

We chose to evaluate the impacts of the high emission scenario (RCP8.5) and the intermediate pathway (RCP4.5) until 2050. The RCP4.5 was chosen instead of RCP6.0 because the predicted emission of RCP8.5 by the end of the century is close to the emission level of the RCP6.0 [70]. Furthermore, these two scenarios are the most commonly used in climate change analysis.

A previous study showed that only temperature (and not precipitation) had a significant effect on Hantavirus infection risk in São Paulo [32]. Thus, we calculated annual and surface temperature (ts) averaged over the state of São Paulo using the two climate change scenarios (RCP4.5 and RCP8.5) and historical experiments. Historical experiments are necessary to calculate temperature anomalies (i.e., calculated with respect to each model´s climatology of the current climate), relative to each model of climate change, being used as baseline. Therefore, 32 general circulation models (GCMs), common to all three experiments (historical, RCP4.5 and RCP8.5), were obtained from the Columbia University Lamont-Doherty Ocean and Climate Physics Data Library (http://strega.ldeo.columbia.edu:81/) for the state of São Paulo. The historical experiment time-series ends in 2005 in most models. Thus, a 30-year climatology was calculated for the period 1976 to 2005 to best match the observational data (20 years—1993–2012) whereas the RCP4.5 and RCP8.5 extends from 2006 to 2050. Although climate-change projections are increasing in spatial resolution, they are still not appropriate for analyzing disease patterns at scales smaller than an area of 250 x 250 km [72]. Therefore, we used the entire state of São Paulo as a basis for climate change scenarios data.

Temperature anomalies for RCP4.5 and RCP8.5 scenarios were calculated individually for each model as the difference between the future temperature predicted and the mean temperature during the 1976–2005 period of the historical experiment. We then calculated the average anomalies of all 32 models, for each scenario (RCP4.5 and RCP8.5) and included this value, in our predictions. Therefore, we ended up with one average of temperature anomalies between 2013 and 2050 of 32 models for the RCP4.5 scenario and one average of temperature anomalies of 32 models for the RCP8.5 scenario, which we used to simulate Hantavirus infection risk from 2013 to 2050. We present the results of our simulated HCPS risk due to mid-century changes in temperature for the period 2040–2050, to diminish uncertainties in climate analysis.

To account for uncertainties in model simulations and time evolution of changes in the climate, we simulated the range of possible outcomes in HCPS risk using the mean ± one standard deviation of all GCMs temperature anomalies (see results and figures in Supplementary material- S2 Fig).

### Data analysis

The probability of Hantavirus infection risk in the state of São Paulo was calculated as a function of landscape, social and climatic factors using a Bayesian model, and is described in detail in [32]. Given that Hantavirus exhibits high host specificity, with each region having different reservoir host species and virus strains [73], Hantavirus transmission risk was modeled separately for Atlantic forest and Cerrado biomes. Although some geographic overlap occurs [74], Araraquara virus (ARAV) is the dominant Hantavirus in Cerrado [74], whereas Juquitiba (JUQV) is the dominant one in Atlantic forest [74]. Municipalities were assigned to Cerrado or Atlantic forest if >50% of their surface area fell inside one of the biome. The biome distribution map was obtained from IBGE (www.ibge.gov.br).

HCPS infection risk was predicted using a Bernoulli distribution and the model (baseline model) contained 7 predictor variables as fixed covariates: proportion of sugarcane, proportion of native vegetation cover, density of native vegetation patches, HDI, mean annual temperature (°C), total annual precipitation (mm), and rural male population >14 years old [32]. Risk was defined as the annual probability of HCPS infection. Municipality was included as a random effect to account for differences among these administrative units that are not captured in the fixed covariates [32]. To facilitate interpretation, all estimated parameters were standardized by centering them on their mean and dividing by two standard deviations [75].

We tested models of raw HPS incidence as well as model residuals for spatial autocorrelation using Moran’s *I*. We used the spatial contiguity matrix based on the Queen´s case neighborhood relation and treated each year separately. This test is commonly used and accepted as a fair evaluation of spatial autocorrelation and dependence [76], especially in disease studies [77, 78]. For all models and most years, we found no spatial autocorrelation, justifying the use of a non-spatial model.

To evaluate changes in Hantavirus infection risk, estimated probability of HCPS infection under current conditions (baseline model) was compared to the predicted probability under five scenarios: two possible future climate change scenarios (RCP4.5 and RCP8.5), one possible sugarcane expansion scenario, and the combinations of each climate scenarios and sugarcane expansion (RCP4.5 + sugarcane; RCP8.5+ sugarcane) (Table 1).

**Table 1:** Average Hantavirus infection risk per municipality, and minimum, maximum, standard deviation and increase in risk values (relative to the baseline model) for the state of São Paulo, considering all 645 municipalities, for the baseline model and each scenario evaluated.

Scenario	Average risk/ municipality	Minimum risk	Maximum risk	Standard deviation	Increase (%)
Current HCPS risk (baseline model)	1.3%	< 0.1%	46.1%	3.4	-
Sugarcane expansion	1.5%	< 0.1%	49.5%	3.6	0.25 (0–6.6)
RCP4.5	1.6%	0.1%	52.3%	3.8	0.35 (0–8.1)
RCP4.5 + sugarcane	1.6%	0.1%	52.4%	3.7	0.35 (0–8.1)
RCP8.5	1.7%	0.1%	52.7%	3.8	0.37 (0.-8.6)
RCP8.5 + sugarcane	1.7%	0.1%	52.8%	3.7	0.37 (0–8.6)

To estimate the predicted probability of HCPS infection under the five future scenarios we used the parameter estimates from the baseline model [32] and used sugarcane and climate data derived for each scenario. We made the simplifying assumption that the biological relationships governing disease transmission would remain largely unchanged over the estimation period. Uncertainty was measured using lower and upper limits of risk estimates for each scenario, derived from the 2.5% and 97.5% quantiles of the baseline model parameters for sugarcane and temperatures. Results are presented in S3 and S4 Figs. The covariates percent of native vegetation cover, number of patches, total annual precipitation, human development index, and people at risk were assumed to be the same as the covariates from the previous year, available from the baseline model (year of 2012), and were kept constant for the predictions. These are reasonable assumptions considering that trends of urban-rural migration in São Paulo are constant [79], deforestation has been drastically reduced in the state [62] and precipitation is not relevant for HCPS risk [32]. Despite the increase in sugarcane mechanization, manual harvest is still necessary and present in some parts of the process [80]. Additionally, skilled workers are replacing unskilled workers, while temporary workers are still being hired at the same rates as before [81]. The number of people employed in sugarcane areas is not diminishing with sugarcane mechanization.

To obtain a clear view of the probability of change in Hantavirus risk, we created a map with the change in infection risk for each scenario that was calculated using the difference between the current Hantavirus risk and the predicted risk for each scenario. We also used model simulations to generate a map of Hantavirus infection risk for the State of São Paulo for each scenario, where Hantavirus infection risk is classified as small (<5%), medium (≥5 and ≤10%), high (≥10 and ≤ 20%) and extremely high (≥20%). We considered that a municipality with a risk higher than 5% should be a target for preventive measures due to the high disease lethality (maps are shown in supplementary material—S5 Fig).

By associating the estimated probability of HCPS infection risk generated for each scenario (baseline model and the five future scenarios) with the at risk population for each municipality (rural men older than 14 years), we predicted current and future human exposure to HCPS. We also calculated the percent increase in the number of people that could be infected in each scenario, by comparing each scenario with the baseline (Table 2).

**Table 2:** Size (indicated by number of people) and dynamic of the population at risk (average, maximum and standard deviation) averaged for all 645 municipalities of state of São Paulo for the current situation and the 5 considered scenarios of sugarcane expansion and climate change.

	Average number of people per municipality at risk for HCPS	Maximum number of people per municipality at risk for HCPS and standard deviation	Increase in the number of people in relation to current risk
**Current HCPS risk**	29	3482 (185)	-
**Sugarcane expansion**	34	3731 (199)	20%
**RCP4.5**	38	3940 (210)	31%
**RCP4.5 + sugarcane**	38	3940 (143)	31%
**RCP8.5**	39	3979 (212)	35%
**RCP8.5+ sugarcane**	39	3979 (144)	35%

## Results

According to our sugarcane expansion scenario, this crop cover will increase to ~30% on average in the state of São Paulo by 2050. Sugarcane area will increase from 26% to 34% in the Cerrado region (11,200 to 14,500 ha) and from 23% to 31% in the Atlantic Forest region (8,200 to 11,100 ha) (S6 Fig).

**Fig 2:**
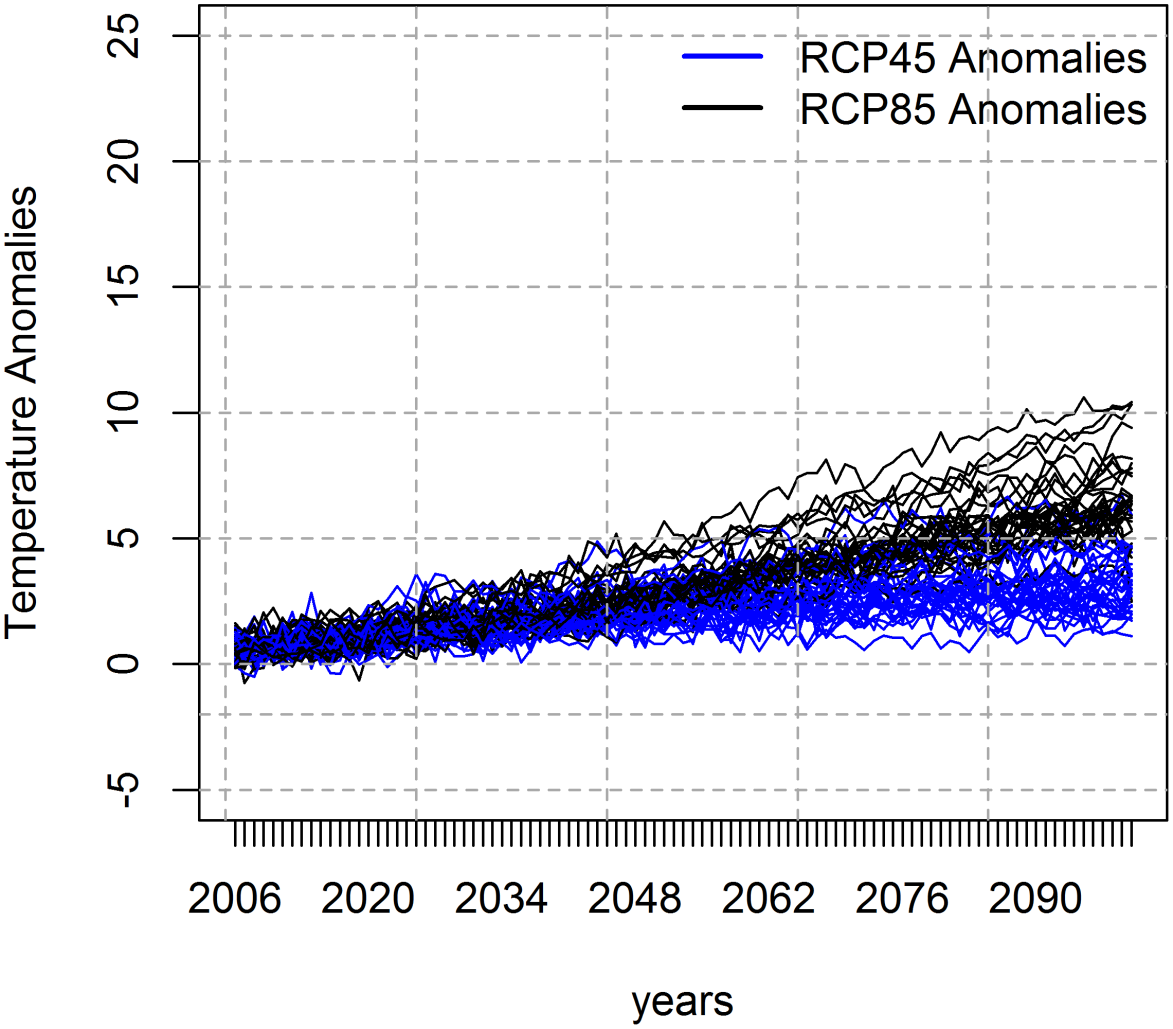
Temperature anomalies. Annual temperature anomalies of the state of São Paulo, from 2006 to 2100, from the 32 RCP4.5 and RCP8.5 models used in the climate change analysis.

Considering climate change scenarios, there is a general consensus among the 32 models evaluated, for both RCP4.5 and RCP8.5 scenarios, in the direction of the projected temperature change for the state of São Paulo, with both experiments presenting increases. Also, RCP8.5 presents a smaller variation and lower standard deviation between the 32 models analyzed than RCP4.5 models, especially from 2013 to 2050 (Fig 2). After 2050, the anomalies of RCP8.5 become larger than those from the RCP4.5 experiment, showing larger increases in temperature anomalies.

**Fig 3:**
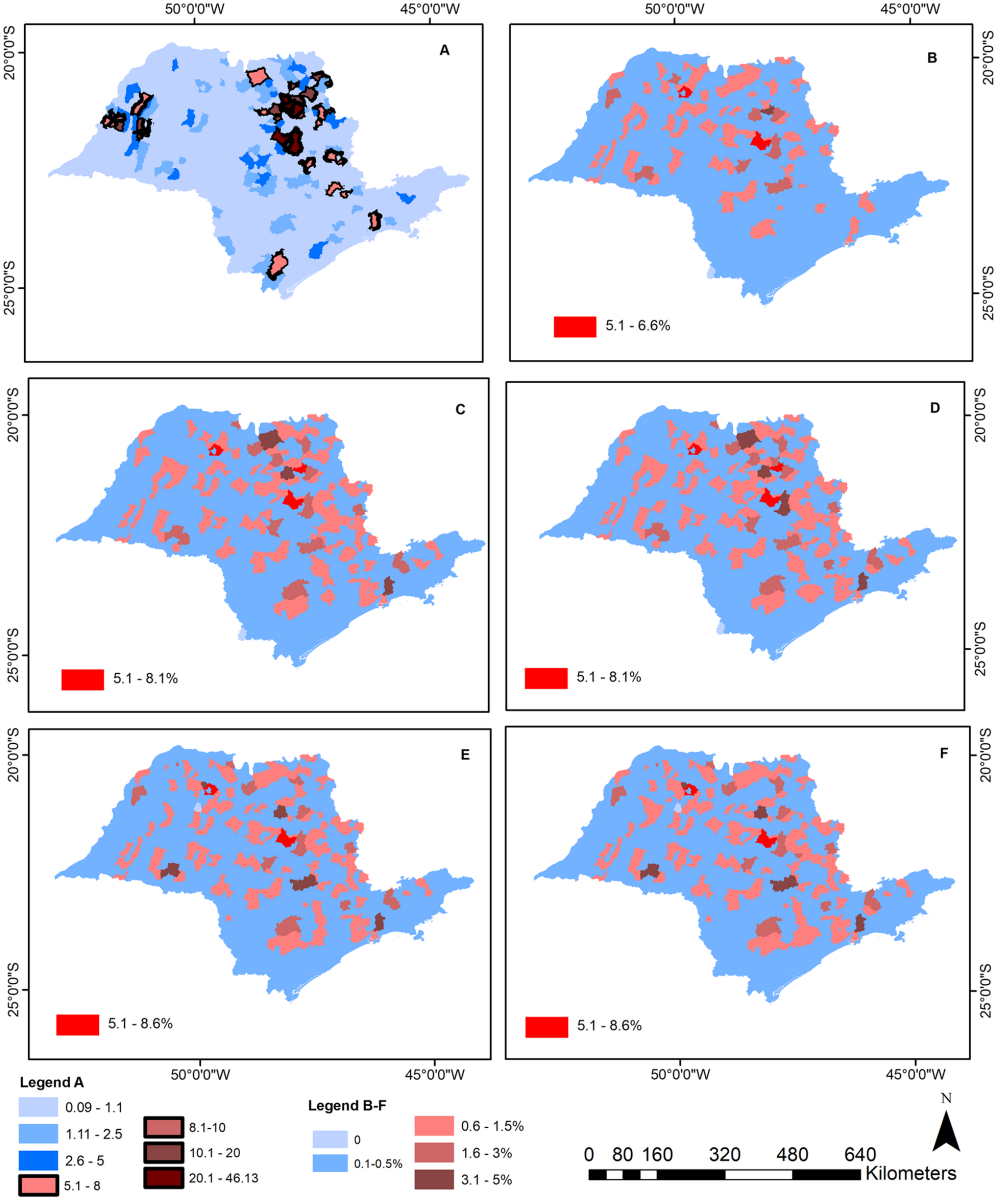
HPS risk maps. Current condition (baseline, A) and probability change in Hantavirus infection risk according to five scenarios: only sugarcane expansion (B), 2040–2050 temperature anomalies from the RCP4.5 (C) and RCP8.5 scenarios (D), combined effect of sugarcane expansion with RCP4.5 (E) and RCP8.5 (F) scenarios. Legend inside each map represents the change in the maximum risk for each scenario. Legend A is associated with map A and legends B-F with maps B to F.

Under current conditions, the state has an average Hantavirus infection risk of 1.3%, with 6% of the municipalities classified as high risk (HCPS ≥ 5%). Hantavirus infection risk increases under all scenarios evaluated (0.25% to 0.37%) (Table 1). Sugarcane expansion is the scenario that predicted the smallest increase in Hantavirus risk, with a 0.25% increase on average. The most pronounced changes are expected to occur in the west and mid-west parts of the state where almost all municipalities exhibit an increase of 1.5% in HCPS infection risk (Fig 3B). Also, sugarcane scenario will lead to a risk greater than 5% for HCPS for about 6.6% of all municipalities (43 municipalities).

Projected temperature anomalies for both climate change scenarios predicted similar average increases of HCPS in the state (0.35% for RCP4.5 and 0.37% for RCP8.5), with larger increases concentrated in the northeast region, but with RCP4.5 predicting smaller increases than RCP8.5 (Fig 3C and 3D). Moreover, there is a significant increase in the risk of infection for some municipalities that already had a high risk, especially in the mid-west region, with HCPS risk reaching 52.3% in RCP4.5 and 52.7% in RCP8.5. Under the RCP4.5 scenario there were 42 municipalities (~6.5% of the state) with a HCPS risk greater than 5%, while under the RCP8.5 scenario there were 44 municipalities (~6.9% of the state). The HCPS risk simulated using the temperature anomalies ± 1 standard deviation showed that the uncertainty of models simulations is small, and that disease risk is similar for all three experiments (mean temperature anomalies, mean temperature anomalies + 1 standard deviation and mean temperature anomalies—1standard deviation). Therefore, our confidence interval of predictions due to temperature change analysis is narrow, showing similar and consistent trends (S2 Fig). Additionally, the confidence interval of risk estimates for most of municipalities is narrow, except for some municipalities, where upper limits are high (S3A and S3B Fig).

When combining climate change scenarios and sugarcane expansion, the average increase and the maximum HCPS risk for the state is the same as under the climate change scenarios alone (RCP4.5 and RCP8.5), showing that there is no additional effects between temperature and sugarcane. However, the increase in Hantavirus risk became more homogeneous throughout the state when considering the combined sugarcane-climate change (Fig 3E and 3F), with the inclusion of ~7% of the municipalities of the state with HCPS risk greater than 5%.

When we consider the number of people that can be infected by HCPS (based on the number of population at risk), the sugarcane expansion scenario alone presents an increase of 20%. For RCP4.5 and RCP8.5 temperature scenarios alone and combined with sugarcane expansion the number of people is the same, presenting an increase of 31% and 35% respectively (Table 2).

## Discussion

In accordance to our predictions, sugarcane expansion and rising temperature will lead to increases in HCPS risk but with relatively weak effects on average. Our results suggest that climate change effects will be more severe than those from sugarcane expansion, and surprisingly, there was no evidence of additive effects of sugarcane and climate on HCPS risk for the state.

The effects of sugarcane expansion and temperature anomalies on HCPS risk were smaller than initially expected, which may have occurred because transmission to humans is complex and involves a number of factors that are not yet fully understood, especially in the tropics. Hantavirus infection rates and prevalence in rodent populations are generally low [34]. Transmission increases in density-dependent fashion with greater intraspecific encounter rates and virus transmission at high rodent densities [7]. Consequently, the virus load in the environment and the human risk of acquiring HCPS also increases at high rodent densities [82]. However, high abundances of reservoirs alone do not guarantee that humans will become infected. To acquire HCPS, human exposure to infected rodents is also necessary, with disease transmission resulting from a combination of human behaviors (i.e., inadequate storage of grains and lack of protective measures) and, density and prevalence of reservoirs. Climate also affects virus survival and aerosolization in the environment [41]. Disease transmission to humans requires that these four main factors interact: an infected rodent; a certain abundance of reservoir rodents to proliferate the infection throughout the rodent population (in which prevalence is generally low); suitable climatic conditions in order to maintain the virus in the environment and allow its aerosolization, and a susceptible human population. Due to these complex dynamics, HCPS transmission to humans is difficult and can be considered as a rare event, with a low number of cases reported each year. However, this increase is extremely relevant given the high lethality rate of the HCPS, which is around 50% in the state.

### Sugarcane expansion and HCPS risk

Our results confirmed previous studies showing that increases in the amount of sugarcane can augment HCPS risk [32, 44, 83]. Our scenario predicted an increase in ~6,000 ha of land occupied with sugarcane on average, for both the Cerrado and Atlantic forest regions, until 2050, forecasting increases of ~15% in HCPS risk. This expansion can be considered small, since the area planted with sugarcane in the state has tripled from 1990 to 2010, increasing from 3,000 to 9,000 ha on average for the entire state [56, 84]. Over this same time period HCPS risk in the state has also increased almost four times (382%) from 0.34 to 1.3%. Therefore, the increase in disease risk, predicted by our model and according to the expansion of sugarcane, is concordant with the historical increase in risk experienced from 1993 to 2012.

This increase in disease risk, without any change in temperature, is on average low, but can be as high as 6.6% in some municipalities. Overall our sugarcane expansion scenario predicts an increase of 20% in the number of people that can acquire HCPS. The main underlying mechanism to explain this pattern is that sugarcane provides a highly nutritious food, leading to increased recruitment and a rapid population growth of rodents [85]. Sugarcane plantations are a suitable habitat for these generalist rodent species, as Hantavirus reservoirs, supporting greater abundances of rodents than other ecosystems, whether natural or agricultural [47], with sugarcane becoming a predominant part of their diets [86].

Land-use changes also indirectly influence local temperature [87] and alter albedo and evapotranspiration, which can directly influence local climate [88]. Sugarcane plantations have cooler temperatures and more moisture than pasture and other crops, being micro-climatically more similar to areas of natural vegetation near to the soil level [87]. This microclimate changes may contribute to the increase in HCPS risk, since it makes sugarcane an even better habitat for rodents. This climate aspect can also affect the indirect path of transmission, extending the time the virus remains infectious in this environment and augmenting HCPS risk, since virus inactivation happens only in dry conditions and above 37°C [41].

### Climate change and HCPS risk

Climate change scenarios predict larger increases in HCPS risk when compared with sugarcane expansion alone. Increases in temperature may be more important than sugarcane expansion, because temperature interacts with disease transmission through multiple mechanisms. Temperature positively affects vegetation growth [36, 89], leading to increases in the abundance of reservoir rodent species, since small mammal populations are food-limited [45]. Temperature also affects reproduction and survival of small rodents [36, 40], which may have a positive or negative effect depending on the magnitude of temperature change [36, 90]. In addition, temperature directly affects process of HCPS transmission, determining virus survival and aerosolization in the environment [41].

There is a lack of studies involving reservoir rodent species and Hantavirus related to HCPS and climate variables, but for HFRS, mild temperatures (10–25°C) are most favorable for breeding of reservoirs rodents [91] and for the time the virus remains infectious in the environment [41]. Increases in temperature lead to greater aerosolization of the virus and higher rates of inhalation by both humans and rodents [92]. In this way, increases in temperature may have a positive effect on reservoir rodent abundance and virus survival and aerosolization until reaching a certain threshold (around 40°0°CC) from where temperature will exert a negative effect.

The relatively low magnitude of the effect of climate change on HCPS risk in our study maybe explained by the fact that temperature anomalies, until 2050, can be considered small and similar for both RCP4.5 and RCP8.5, with larger increases being observed only after 2050. Nevertheless, it is important to highlight that increases in temperature anomalies lead to increase in HCPS risk, though small, in all the 645 municipalities of the state of São Paulo. Therefore, higher increases for disease risk are expected after 2050 if carbon emissions are not controlled and climate change mitigation actions are not successful.

### Combined effect of climate change and sugarcane on HCPS risk

Individual evaluation of climate and sugarcane could have resulted in a better understanding of the individual contributions of each factor on disease risk. However, evaluating these scenarios together is a more realistic approach, given that they will occur and act together. Sugarcane expansion and temperature anomalies together showed no additionality, predicting the same average increase in HCPS risk when compared to climate change scenarios alone. This may have happened because there are multiple mechanisms through which temperature influences HCPS risk, some of which overlaps sugarcane mechanisms (i.e., effect on rodent densities). Particularly, even in conditions where rodent abundances and prevalence are high, if temperature conditions are not ideal for virus survival and aerosolization, transmission to humans will not occur. Therefore, the ability of the virus to survive outside the host is critical for the transmission within rodent populations and to humans, with temperature being one of the determining factors of this survival. This effect, may have contributed to the lack of additionality between temperature and sugarcane, since sugarcane effects will only occur when temperature conditions are also adequate.

### Conservation and public health implications

Land cover and land use change are at the origin of the outbreaks of Hantavirus and can also be an important component to reduce or mitigate its spread. Given that temperature increase will lead to increases in HCPS risk, forest restoration can be an alternative to attenuate the effects of higher temperature on HCPS risk for three main reasons. First, forest regrowth, especially in tropical regions, can sequester atmospheric carbon, absorbing about 30% of all CO_2_ emissions from fossil fuel burning and net deforestation [88, 93], contributing to climate change mitigation. Second, forest regeneration can mitigate the creation of warmer and drier climates in agricultural systems [88], reducing the ideal conditions for hantavirus survival. Third, increasing forest cover could also reduce HCPS risk arising from sugarcane expansion, since it would lead to increased suitable habitat for habitat specialist species, leading to a more diverse community, with decreased abundance of habitat generalist species [94], such as hantavirus reservoir species.

We note that the use of ethanol from sugarcane as a gas substitute leads to a very important reduction in greenhouse gases emissions, which can reach up to 85% [95, 96]. This is mostly due to the fact that they replace fossil fuels [97], and sequester carbon through the growth of the feedstock [98, 99], especially when pastures are converted to sugarcane fields and managed without fire [95]. The use of ethanol from sugarcane, produced in landscapes with a large amount of connected forest cover, could ameliorate disease risk, since it would increase diversity community, diminishing reservoir rodent abundance, and would contribute to climate mitigation.

Public health costs will also increase under the expected increase in temperature and sugarcane expansion. At least part of these costs should be factored into sugarcane production, including expenditures associated with rodent control, education and preventive campaigns targeting how to avoid virus inhalation and contact with infected rodents excreta. These control measures are likely to yield additional benefits since rodents are considered major pests of this crop [86], leading to a loss of 825.000 tons of sugarcane in one year in India [100]. States and municipalities considering sugarcane expansion should also plan for costs involved with educational campaigns and preventive measures, for example, educating workers and residents from rural areas about how to avoid Hantavirus inhalation and contact with infect rodents excreta. This could be crucial to avoid disease propagation to places where HCPS risk is currently low or absent. This type of information should be incorporated into the costs of land use management. Sugarcane expansion can provide a solution to one specific problem, such as supplying the oil market, but can on the other hand create a human health problem by increasing risks of acquiring HCPS.

Our results reinforce the links between climate change and rises in incidence of diseases, such as Lyme, West Nile Virus and *Echinococcus* [101–103]. These findings should be considered as an additional argument to encourage governments, companies and citizens to sign agreements and start massive campaigns in order to mitigate climate change impacts.

### Final remarks

Our scenarios of future sugarcane expansion and climate change RCP4.5 and RCP8.5 predicted a low but significant increase in HCPS risk in the state of São Paulo by 2050. Despite the lack of additive effects of sugarcane and climate in HCPS risk, we suggest that prevention and mitigation actions should focus on land use planning and forest restoration programs, and by concentrating healthcare effort in areas that are predicted to be at higher HCPS risk and have a high variation in the confidence interval.

To better explore the underlying mechanisms of the observed pattern, we suggest future studies should test the effects of sugarcane production, temperature, and moisture on reservoir rodent population dynamics and on virus survival and aerosolization. Understanding those relationships is crucial to better understand HCPS transmission dynamics in different environments and situations, which is important for the effective design of preventive health strategies.

## Supporting information

S1 Table Predictors. Predictor variables included in the baseline model, years in which data is available, source data and how we modeled this information with disease data.
(DOCX)

S1 Fig The AZA. The Agro-environmental Zoning for the sugar and Alcohol Industry (AZA). The suitability classification is based on edapho-climate conditions and biodiversity protection. Source: Environment Secretary of the state of São Paulo.
(DOCX)

S2 Fig HCPS risk maps with ± standard deviation. Map of change in Hantavirus infection risk according to four scenarios: (A) average temperature anomalies + 1 standard deviation of RCP4.5 (B) average of temperature anomalies—1 standard deviation of RCP4.5 (C) average of temperature anomalies + 1 standard deviation of RCP8.5; (D) average of temperature anomalies—1 standard deviation of RCP8.5. The risk predicted by the average temperature anomalies ± 1 standard deviation, for both RCP4.5 and RCP8.5 scenarios, showed similar patterns of trend, with both predicting the same average of increase for São Paulo state (0.36%) and a small difference in the maximum risk for some municipalities (6.8 to 7.11 for RCP4.5; and 7.37 and 7.45 for RCP8.5). They also show a similar pattern of increase with the risk predicted by the anomalies averaged of the 32 models, indicating a small range of possibilities in the predicted HPS risk and a large confidence in our predictions.
(DOCX)

S3 Fig HCPS risk lower limits. Map of lower limits of Hantavirus infection risk according to the five scenarios evaluated: (A) sugar cane expansion, (B) temperature anomalies of RCP4.5, (C) and RCP8.5 scenarios, (D) RCP4.5 and RCP8.5 scenarios combined with sugar cane expansion (D and E, respectively).
(DOCX)

S4 Fig HCPS risk upper limits. Map of upper limits of Hantavirus infection risk according to the five scenarios evaluated: (A) sugar cane expansion, (B) temperature anomalies of RCP4.5, (C) and RCP8.5 scenarios, (D) RCP4.5 and RCP8.5 scenarios combined with sugar cane expansion (D and E, respectively). Local values (municipalities) are indicated in each map, as well as maximum values for HCPS risk.
(DOCX)

S5 Fig HCPS risk maps. Map of Hantavirus infection risk according to current condition (baseline, A) and five scenarios: sugar cane expansion (B), temperature anomalies of RCP4.5 (C) and RCP8.5 scenarios (D); RCP4.5 and RCP8.5 scenarios combined with sugar cane expansion (E and F, respectively). Local values (municipalities) are indicated in each map, as well as maximum values for HCPS risk.
(DOCX)

S6 Fig Mean sugarcane cover. Mean sugarcane cover (in %) for municipalities with Cerrado and Atlantic forest vegetation in 2012 (“current”) and 2050 according to our model expansion scenario (potential). Black points represent the means, and horizontal bars represent standard errors.
(DOCX)
