# Supplementary material for: Climate change and sugarcane expansion increase Hantavirus infection risk
Source: PLoS Negl Trop Dis. 2017 Jul 20;11(7):e0005705. doi: 10.1371/journal.pntd.0005705 (PMC5519001; doi:10.1371/journal.pntd.0005705)
Supplement: S1 Table — Predictor variables included in the baseline model, years in which data is available, source data and how we modeled this information with disease data. (DOCX) [file pntd.0005705.s001.docx]

Climate change and sugarcane expansion increase Hantavirus infection risk

Paula Ribeiro Prist, María Uriarte, Katia Fernandes, Jean Paul Metzger

**Supporting information**

S1 Table. Predictor variables included in the baseline model, years in which data is available, source data and how we modeled this information with disease data.

| *Predictor variable* | *Description* | *Years available* | *Modeling with disease information* | *Source data* |
| --- | --- | --- | --- | --- |
| People at risk | Number of men older than 14 years employed/ live in agricultural areas | 1996/2006 | 1996 – 1993 to 2001  2006 – 2002 to 2012 | IBGE |
| HDI | Human Development Index | 1991/2000/2010 | 1991 – 1993 to 1998  2000 – 1999 to 2005  2010 – 2006 to 2012 | IBGE |
| Forest | Percentage the landscape in natural vegetation cover | 2000/2010 | 2000 – 1993 to 2001  2010 – 2002 to 2012 | São Paulo state Forest Inventory |
| NP | Number of patches in the landscape | 2000/2010 | 2000 – 1993 to 2001  2010 – 2002 to 2012 | São Paulo state Forest Inventory |
| Sugarcane | Percent of municipality occupied by sugar cane | 1993 to 2012 | 1993 to 2012 (data presented yearly) | Agricultural Census of Institute of Agricultural Economics |
| Total Precipitation | Total annual precipitation | 1993 to 2012 | 1993 to 2012 (data presented yearly) | Climate Hazards Group Infrared Precipitation with Stations |
| Mean Temperature | Mean annual temperature per year | 1993 to 2012 | 1993 to 2012 (data presented yearly) | National Centers for Environmental Prediction |
