# Supplementary material for: Climate change and sugarcane expansion increase Hantavirus infection risk
Source: PLoS Negl Trop Dis. 2017 Jul 20;11(7):e0005705. doi: 10.1371/journal.pntd.0005705 (PMC5519001; doi:10.1371/journal.pntd.0005705)
Supplement: S1 Fig — The Agro-environmental Zoning for the sugar and Alcohol Industry (AZA). The suitability classification is based on edapho-climate conditions and biodiversity protection. Source: Environment Secretary of the state of São Paulo. (DOCX) [file pntd.0005705.s002.docx]

Climate change and sugarcane expansion increase Hantavirus infection risk

Paula Ribeiro Prist, María Uriarte, Katia Fernandes, Jean Paul Metzger

**Supporting information**


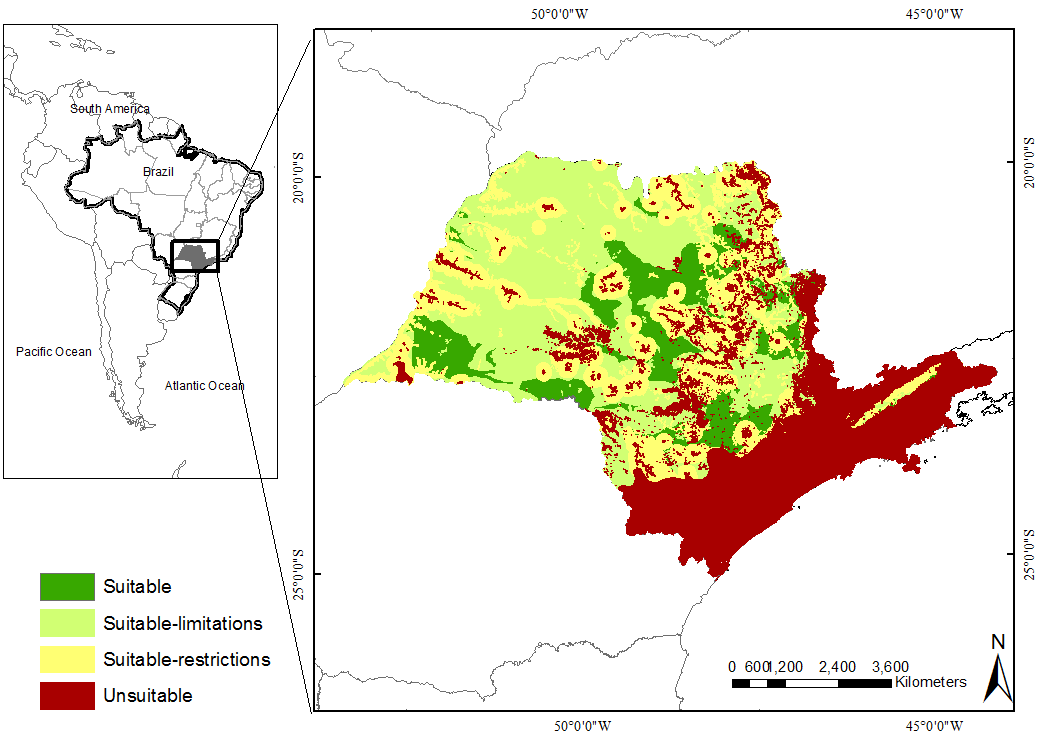


S1 Fig. The Agro-environmental Zoning for the sugar and Alcohol Industry (AZA). The suitability classification is based on edapho-climate conditions and biodiversity protection. Source: Environment Secretary of the state of São Paulo.

AZA divides the State in four zones, according to their suitability for sugarcane: suitable areas (favorable edaphic and climatic conditions, with no environmental constraints); suitable areas with environmental limitations (favorable edaphic and climatic conditions, but includes some protected areas; simple measures of environmental regulation and agricultural management are necessary); suitable areas with environmental restrictions (favorable edaphic and climatic conditions, but include buffer zones of Protected Areas, requiring complex measures of environmental preservation and protection to fauna and flora); and unsuitable areas (unfavorable edaphic and climatic conditions; slope higher than 20%, which prevent mechanization). This zoning was developed using multiple data: climate, air quality, slope, soil, water availability and water quality, protected areas and important fragments for biodiversity connectivity (São Paulo 2008). We considered all three zones of suitable areas as suitable for sugarcane expansion, because it simulates an occupation pattern that is already happening in the state of São Paulo: 50% of the new areas occupied with sugarcane are in suitable areas with environmental limitations, 30% occur in suitable areas with environmental restrictions, and 18% in suitable areas; while 0.8% occur in unsuitable areas (Gomes and Montaño 2012).

References

Gomes PM, Montaño M (2012) A Expansão da Cana-de-Açúcar e o Zoneamento Agroambiental do setor Sucroalcooleiro do estado de São Paulo. 1° Congresso Brasileiro de Avaliação de Impacto. Available at [download 23 July 2015]: http://avaliacaodeimpacto.org.br/wp-content/uploads/2012/10/068_zoneamento.pdf
