# Supplementary material for: Climate change and sugarcane expansion increase Hantavirus infection risk
Source: PLoS Negl Trop Dis. 2017 Jul 20;11(7):e0005705. doi: 10.1371/journal.pntd.0005705 (PMC5519001; doi:10.1371/journal.pntd.0005705)
Supplement: S2 Fig — Map of change in Hantavirus infection risk according to four scenarios: (A) average temperature anomalies + 1 standard deviation of RCP4.5 (B) average of temperature anomalies—1 standard deviation of RCP4.5 (C) average of temperature anomalies + 1 standard deviation of RCP8.5; (D) average of temperature anomalies—1 standard deviation of RCP8.5. The risk predicted by the average temperature anomalies ± 1 standard deviation, for both RCP4.5 and RCP8.5 scenarios, showed similar patterns of trend, with both predicting the same average of increase for São Paulo state (0.36%) and a small difference in the maximum risk for some municipalities (6.8 to 7.11 for RCP4.5; and 7.37 and 7.45 for RCP8.5). They also show a similar pattern of increase with the risk predicted by the anomalies averaged of the 32 models, indicating a small range of possibilities in the predicted HPS risk and a large confidence in our predictions. (DOCX) [file pntd.0005705.s003.docx]

Climate change and sugarcane expansion increase Hantavirus infection risk

Paula Ribeiro Prist, María Uriarte, Katia Fernandes, Jean Paul Metzger

**Supporting information**


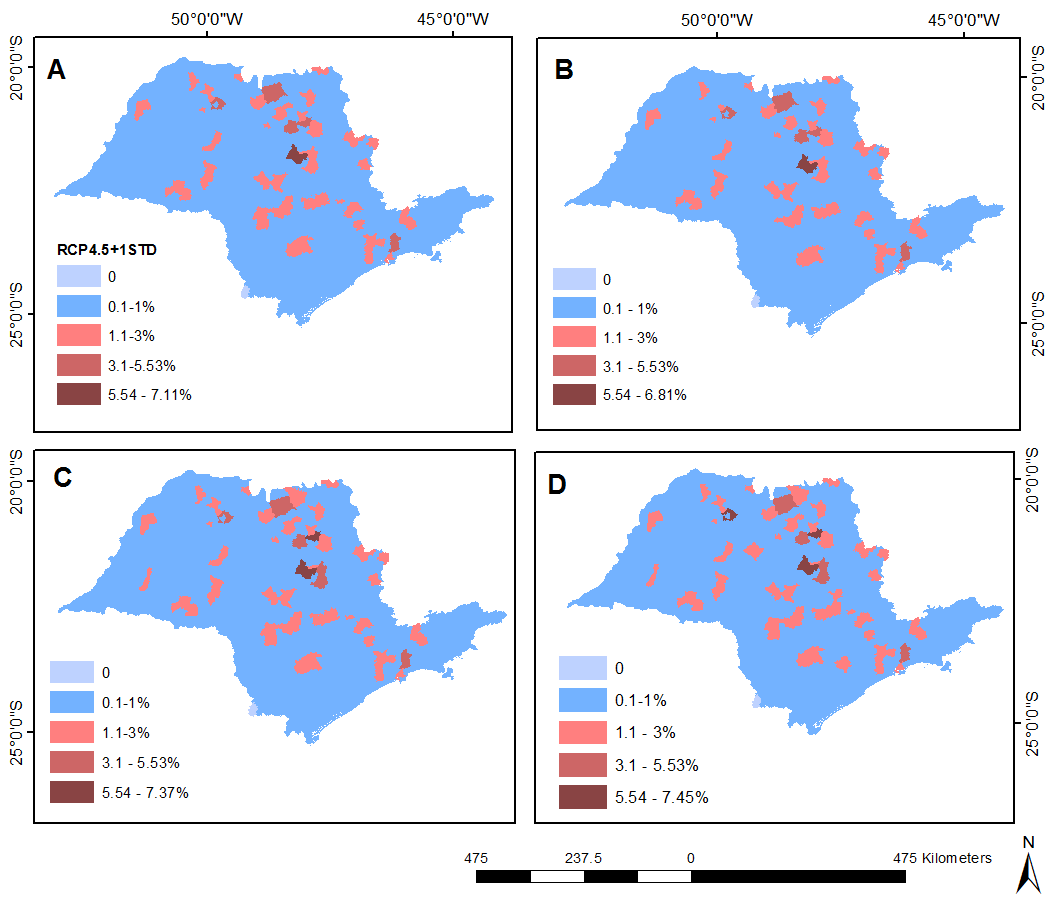


S2 Fig. Map of change in Hantavirus infection risk according to four scenarios: (A) average temperature anomalies + 1 standard deviation of RCP4.5 (B) average of temperature anomalies - 1 standard deviation of RCP4.5 (C) average of temperature anomalies + 1 standard deviation of RCP8.5; (D) average of temperature anomalies - 1 standard deviation of RCP8.5. The risk predicted by the average temperature anomalies ± 1 standard deviation, for both RCP4.5 and RCP8.5 scenarios, showed similar patterns of trend, with both predicting the same average of increase for São Paulo state (0.36%) and a small difference in the maximum risk for some municipalities (6.8 to 7.11 for RCP4.5; and 7.37 and 7.45 for RCP8.5). They also show a similar pattern of increase with the risk predicted by the anomalies averaged of the 32 models, indicating a small range of possibilities in the predicted HPS risk and a large confidence in our predictions.
