# Supplementary material for: Climate change and sugarcane expansion increase Hantavirus infection risk
Source: PLoS Negl Trop Dis. 2017 Jul 20;11(7):e0005705. doi: 10.1371/journal.pntd.0005705 (PMC5519001; doi:10.1371/journal.pntd.0005705)
Supplement: S3 Fig — Map of lower limits of Hantavirus infection risk according to the five scenarios evaluated: (A) sugar cane expansion, (B) temperature anomalies of RCP4.5, (C) and RCP8.5 scenarios, (D) RCP4.5 and RCP8.5 scenarios combined with sugar cane expansion (D and E, respectively). (DOCX) [file pntd.0005705.s004.docx]

Climate change and sugarcane expansion increase Hantavirus infection risk

Paula Ribeiro Prist, María Uriarte, Katia Fernandes, Jean Paul Metzger

**Supporting information**


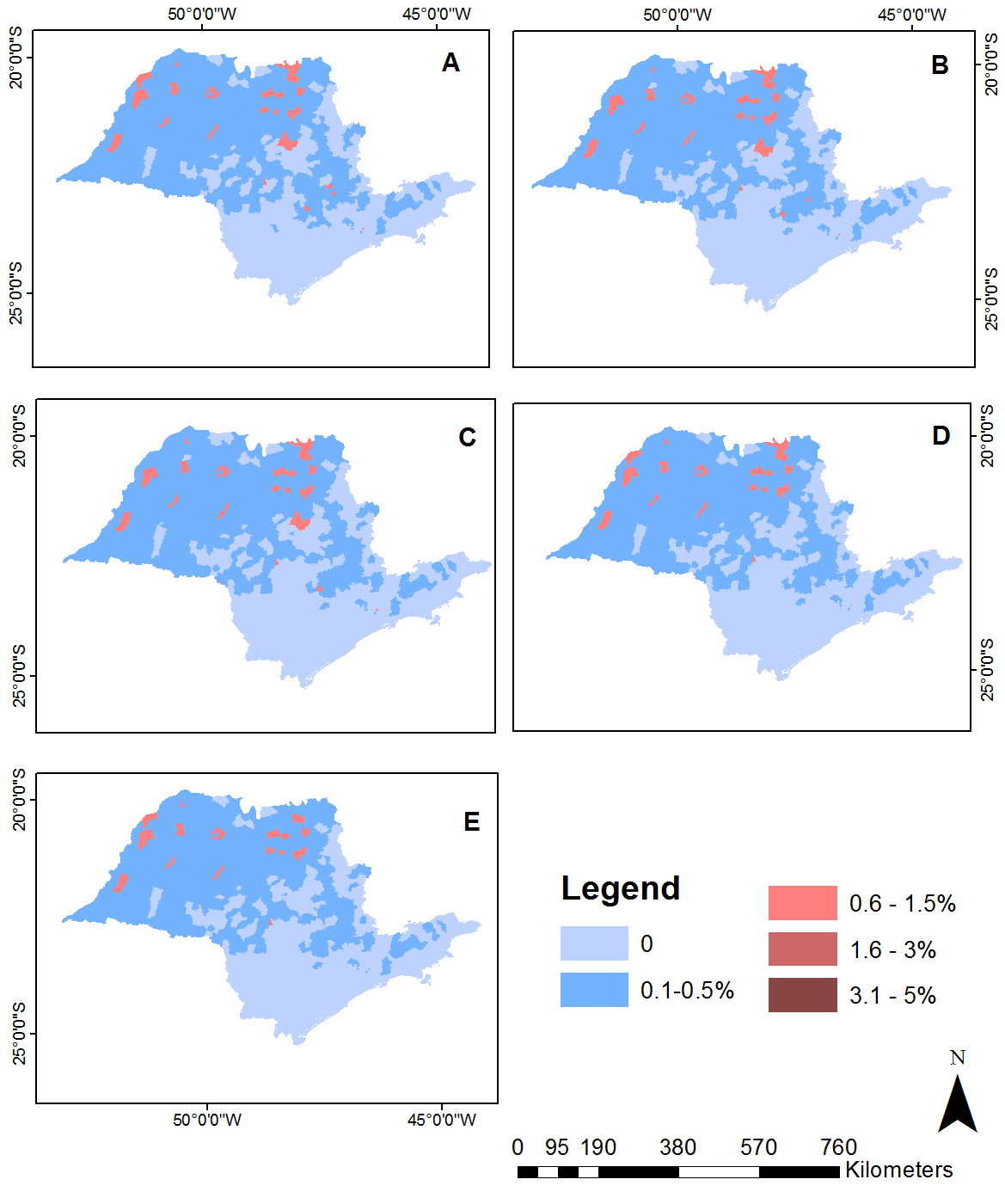


S3 Fig. Map of lower limits of Hantavirus infection risk according to the five scenarios evaluated: (A) sugar cane expansion, (B) temperature anomalies of RCP4.5, (C) and RCP8.5 scenarios, (D) RCP4.5 and RCP8.5 scenarios combined with sugar cane expansion (D and E, respectively).
