# Supplementary material for: Climate change and sugarcane expansion increase Hantavirus infection risk
Source: PLoS Negl Trop Dis. 2017 Jul 20;11(7):e0005705. doi: 10.1371/journal.pntd.0005705 (PMC5519001; doi:10.1371/journal.pntd.0005705)
Supplement: S4 Fig — Map of upper limits of Hantavirus infection risk according to the five scenarios evaluated: (A) sugar cane expansion, (B) temperature anomalies of RCP4.5, (C) and RCP8.5 scenarios, (D) RCP4.5 and RCP8.5 scenarios combined with sugar cane expansion (D and E, respectively). Local values (municipalities) are indicated in each map, as well as maximum values for HCPS risk. (DOCX) [file pntd.0005705.s005.docx]

Climate change and sugarcane expansion increase Hantavirus infection risk

Paula Ribeiro Prist, María Uriarte, Katia Fernandes, Jean Paul Metzger

**Supporting information**


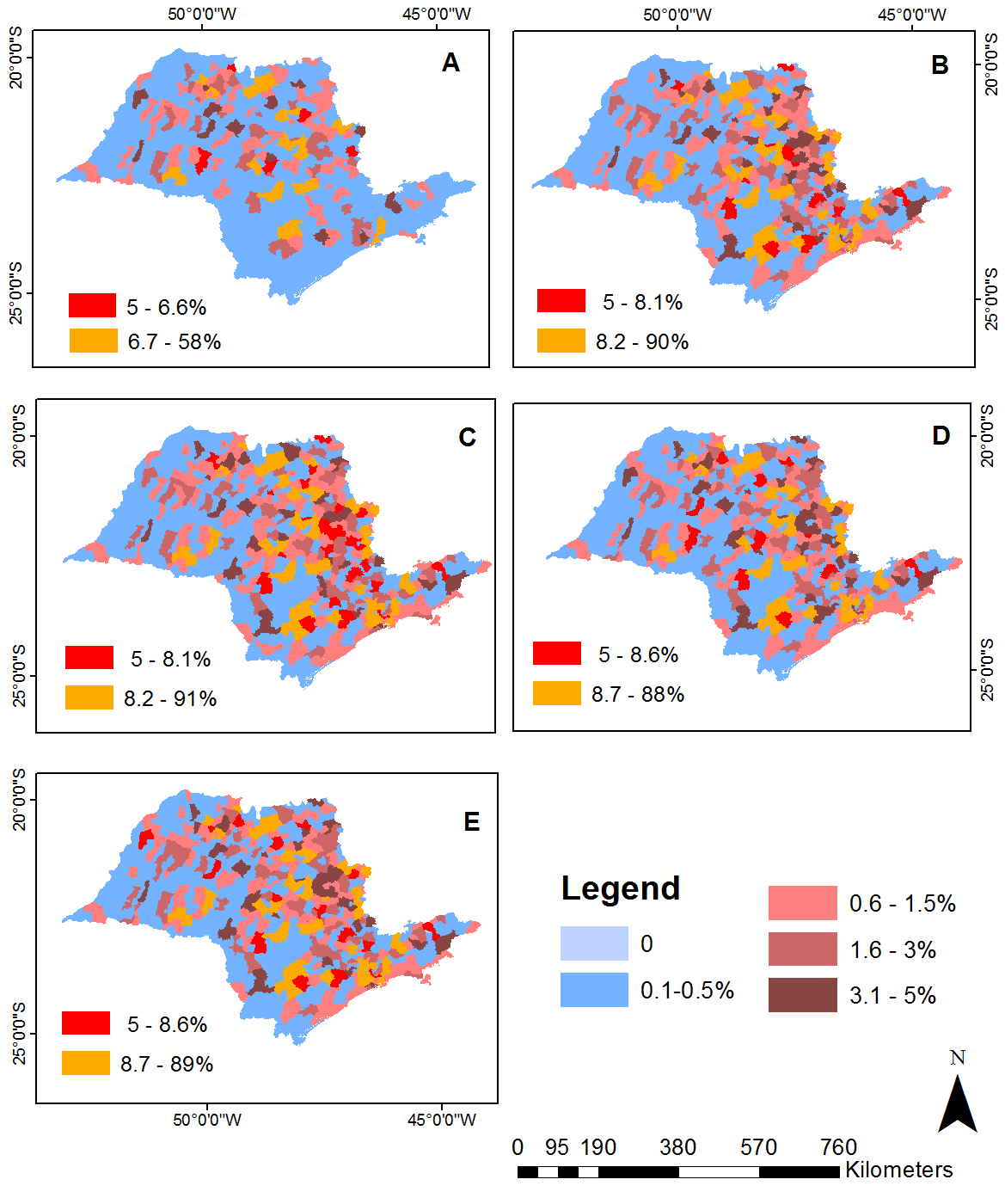


S4 Fig. Map of upper limits of Hantavirus infection risk according to the five scenarios evaluated: (A) sugar cane expansion, (B) temperature anomalies of RCP4.5, (C) and RCP8.5 scenarios, (D) RCP4.5 and RCP8.5 scenarios combined with sugar cane expansion (D and E, respectively). Local values (municipalities) are indicated in each map, as well as maximum values for HCPS risk.
