# Supplementary material for: Climate change and sugarcane expansion increase Hantavirus infection risk
Source: PLoS Negl Trop Dis. 2017 Jul 20;11(7):e0005705. doi: 10.1371/journal.pntd.0005705 (PMC5519001; doi:10.1371/journal.pntd.0005705)
Supplement: S5 Fig — Map of Hantavirus infection risk according to current condition (baseline, A) and five scenarios: sugar cane expansion (B), temperature anomalies of RCP4.5 (C) and RCP8.5 scenarios (D); RCP4.5 and RCP8.5 scenarios combined with sugar cane expansion (E and F, respectively). Local values (municipalities) are indicated in each map, as well as maximum values for HCPS risk. (DOCX) [file pntd.0005705.s006.docx]

Climate change and sugarcane expansion increase Hantavirus infection risk

Paula Ribeiro Prist, María Uriarte, Katia Fernandes, Jean Paul Metzger

**Supporting information**


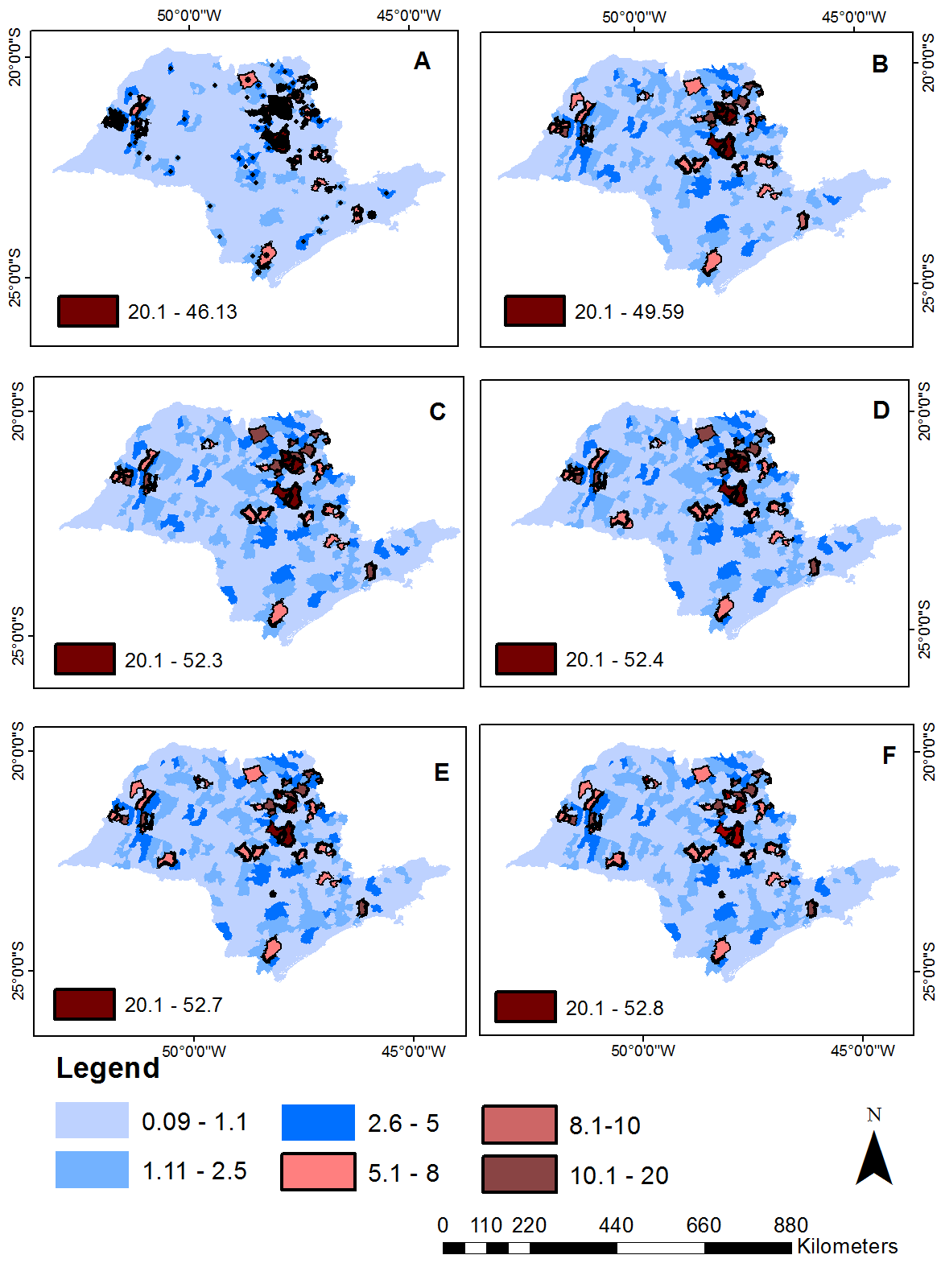


S5 Fig. Map of Hantavirus infection risk according to current condition (baseline, A) and five scenarios: sugar cane expansion (B), temperature anomalies of RCP4.5 (C) and RCP8.5 scenarios (D); RCP4.5 and RCP8.5 scenarios combined with sugar cane expansion (E and F, respectively). Local values (municipalities) are indicated in each map, as well as maximum values for HCPS risk.
