# Supplementary material for: Climate change and sugarcane expansion increase Hantavirus infection risk
Source: PLoS Negl Trop Dis. 2017 Jul 20;11(7):e0005705. doi: 10.1371/journal.pntd.0005705 (PMC5519001; doi:10.1371/journal.pntd.0005705)
Supplement: S6 Fig — Mean sugarcane cover (in %) for municipalities with Cerrado and Atlantic forest vegetation in 2012 (“current”) and 2050 according to our model expansion scenario (potential). Black points represent the means, and horizontal bars represent standard errors. (DOCX) [file pntd.0005705.s007.docx]

Climate change and sugarcane expansion increase Hantavirus infection risk

Paula Ribeiro Prist, María Uriarte, Katia Fernandes, Jean Paul Metzger

**Supporting information**


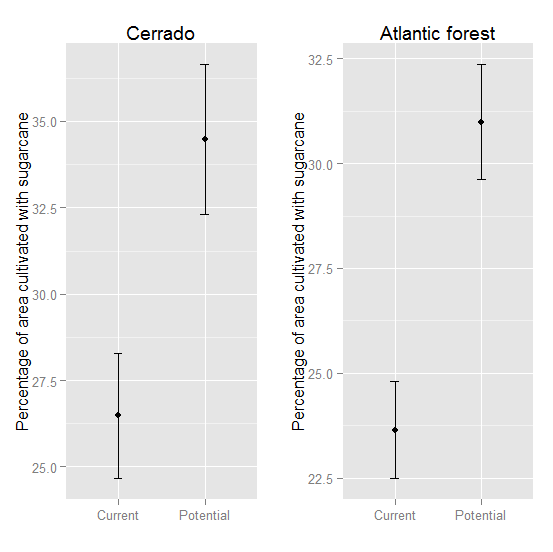


S6 Fig. Mean sugarcane cover (in %) for municipalities with Cerrado and Atlantic forest vegetation in 2012 (“current”) and 2050 according to our model expansion scenario (potential). Black points represent the means, and horizontal bars represent standard errors.
